# Functional Characterization of Maize *ZmMTP1-1* and *ZmMTP1-2* Reveals Their Roles in Cd Tolerance

**DOI:** 10.3390/plants15060941

**Published:** 2026-03-19

**Authors:** Wenyu Li, Jialun Zhu, Yanrui Liu, Jing Ma, Yingqi Qu, Wei Yang, Chengbo Zhang, Cong Li, Yanye Ruan, Xingxing Dong, Shuang Yang, Sidra, Yijun Tang, Xiaomei Dong, Jinjuan Fan

**Affiliations:** 1 College of Bioscience and Biotechnology, Shenyang Agricultural University, Shenyang 110866, China; 2 Shenyang City Key Laboratory of Maize Genomic Selection Breeding, Shenyang 110866, China; 3 Shenyang Rural Revitalization and Development Center, Shenyang 110034, China; 4 Department of Resources and Environment, Zunyi Normal College, Zunyi 563006, China

**Keywords:** maize, metal tolerance protein, ZmMTPs, cadmium stress, heavy metal tolerance, antioxidant defense

## Abstract

Cadmium (Cd) contamination severely threatens crop productivity and food safety, particularly in maize (*Zea mays* L.), which exhibits relatively high capacities for metal uptake and translocation. Metal tolerance proteins (MTPs) play essential roles in metal homeostasis and detoxification; however, the functions of maize MTP under Cd stress remain poorly understood. In this study, a comprehensive expression analysis of the maize MTP gene family revealed that two Zn-CDF members, *ZmMTP1-1* and *ZmMTP1-2*, displayed the strongest and most consistent transcriptional induction in response to Cd stress, especially in roots. Phylogenetic and structural analyses confirmed that both genes are closely related to MTP1 homologs from other plant species, while exhibiting distinct gene structures and regulatory features. Functional characterization in transgenic *Arabidopsis thaliana* demonstrated that overexpression of *ZmMTP1-1* or *ZmMTP1-2* significantly enhanced tolerance to Cd and Zn stress, as reflected by improved seed germination, root growth, survival, and biomass accumulation. Enhanced metal tolerance was associated with elevated antioxidant enzyme activities, reduced oxidative damage, and coordinated upregulation of endogenous metal transporter genes. Moreover, heterologous expression of *ZmMTP1-1* in yeast further supported its conserved role in Cd tolerance. Collectively, these findings indicate that *ZmMTP1-1* and *ZmMTP1-2* contribute to Cd detoxification through coordinated metal transport and stress-response pathways, providing potential genetic resources for improving heavy metal tolerance in maize.

## 1. Introduction

Cadmium (Cd) is a highly toxic and non-essential heavy metal that poses a serious threat to agricultural productivity and food safety worldwide. Due to its high mobility in soils and strong bioavailability, Cd can be readily absorbed by plant roots and translocated to aerial tissues, where it disrupts cellular homeostasis, inhibits growth, and ultimately accumulates in edible organs [1,2]. In maize (*Zea mays* L.), one of the most important cereal crops globally, Cd contamination not only reduces yield but also increases the risk of Cd entry into the human food chain, highlighting the urgent need to elucidate the molecular mechanisms underlying Cd tolerance and detoxification in this species.

Plants have evolved sophisticated mechanisms to cope with excess heavy metals, including restriction of metal uptake, chelation by organic ligands, vacuolar sequestration, and activation of antioxidant defense systems [3,4]. Among these mechanisms, metal transporters play central roles in regulating metal uptake, distribution, and compartmentalization. Several transporter families have been implicated in heavy metal homeostasis, such as heavy metal ATPases (HMAs), natural resistance-associated macrophage proteins (NRAMPs), zinc–iron permeases (ZIPs), pleiotropic drug resistance proteins (PDRs), and cation diffusion facilitators (CDFs), also known as metal tolerance proteins (MTPs) [5,6]. Coordinated regulation of these transporters is essential for maintaining metal ion balance while minimizing metal-induced toxicity.

The MTP family belongs to the CDF superfamily and is characterized by six transmembrane domains and a histidine-rich cytosolic loop between transmembrane domains IV and V, which is critical for metal binding and transport activity [7]. Based on substrate specificity, plant MTPs are generally classified into three subfamilies: Zn-CDF, Zn/Fe-CDF, and Mn-CDF [8]. Functional studies in model plants have demonstrated that MTPs are involved in the transport and sequestration of Zn^2+^, Fe^2+^, Mn^2+^, and Cd^2+^, thereby contributing to metal tolerance and homeostasis. For example, *Arabidopsis thaliana AtMTP1* functions as a vacuolar Zn transporter and confers tolerance to excess Zn and Cd [9] (Kobae et al., 2004), while rice *OsMTP1* plays an important role in Zn and Cd detoxification by mediating metal sequestration into vacuoles [10,11].

Despite increasing evidence for the importance of MTPs in metal tolerance, their roles in maize remain incompletely understood. Genome-wide analyses have identified multiple MTP genes in maize; however, functional characterization of individual members, particularly those belonging to the Zn-CDF subfamily, is still limited. Moreover, maize exhibits species-specific physiological traits, such as high transpiration rates and strong metal uptake capacity, suggesting that maize MTPs may possess unique regulatory features and functional properties compared with their orthologs in rice and Arabidopsis [12]. Elucidating the functions of maize MTPs is therefore essential for understanding Cd detoxification strategies in this crop.

In this study, we performed a comprehensive analysis of the maize MTP gene family and classified its members into Zn-CDF, Zn/Fe-CDF, and Mn-CDF subfamilies based on phylogenetic relationships with Arabidopsis MTPs. By combining transcriptome profiling and qRT-PCR analysis under Cd stress, we identified two Zn-CDF members, *ZmMTP1-1* and *ZmMTP1-2*, that exhibited strong and sustained Cd-induced expression. We further investigated their biological functions through heterologous expression in yeast and overexpression in *A. thaliana*. Our results demonstrate that *ZmMTP1-1* and *ZmMTP1-2* play critical roles in Cd and Zn tolerance by coordinating metal transport, antioxidant defense, and stress-responsive regulatory networks. These findings provide new insights into the molecular basis of heavy metal detoxification in maize and identify promising genetic targets for the development of Cd-tolerant maize cultivars.

## 2. Materials and Methods

### 2.1. Plant Materials and Growth Conditions

Maize inbred line B73 was used for gene expression analysis. Seeds were germinated and grown in pots containing soil collected from the experimental field of Shenyang Agricultural University. Plants were cultivated under controlled conditions (27 ± 3 °C, 70% relative humidity, 600 μmol·m^−2^·s^−1^ photosynthetic photon flux density, 16/8 h day/night cycle). At the three-leaf stage, seedlings were subjected to cadmium (Cd) treatment by irrigating roots with 100 μmol·L^−1^ CdCl_2_ solution. Root and leaf samples were harvested at 0, 6, 12, and 24 h after treatment, immediately frozen in liquid nitrogen, and stored at −80 °C for RNA extraction. Three independent biological replicates were collected for each time point.

### 2.2. RNA Extraction and Quantitative Real-Time PCR (qRT-PCR) Analysis in Maize

Total RNA was isolated from maize roots and leaves using TRIzol reagent (Invitrogen, Carlsbad, CA, USA) according to the manufacturer’s instructions. First-strand cDNA was synthesized using a FastKing RT Kit (Tiangen Biotech, Beijing, China). Quantitative real-time PCR (qRT-PCR) was performed using 2× SuperReal PreMix Plus on a Bio-Rad Laboratories, Hercules, CA, USA real-time PCR system. Gene-specific primers for ZmMTP family members were designed and are listed in Table S1. *ZmActin* (*Zm00001d013367*) was used as an internal reference gene. Relative expression levels were calculated using the 2^−ΔΔCT^ method. Three biological replicates and three technical replicates were included for each sample.

### 2.3. Phylogenetic and Promoter Analysis of ZmMTP1-1 and ZmMTP1-2

Protein sequences of ZmMTPs, and MTP homologs from other plant species were retrieved from public databases. Multiple sequence alignments and phylogenetic tree construction were performed using MEGA 12.0 software. Promoter sequences (2 kb upstream of the start codon) were analyzed for cis-regulatory elements using the PlantCARE database (Version 24.0, https://bioinformatics.psb.ugent.be/webtools/plantcare/html/ (accessed on 12 March 2026), Ghent University, Ghent, Belgium). For protein interaction network construction, protein IDs were input into the Search Tool for the Retrieval of Interacting Genes/Proteins (STRING, Version 11.5, https://cn.string-db.org/ (accessed on 12 March 2026), Swiss Institute of Bioinformatics, Lausanne, Switzerland) database.

### 2.4. Protein–Protein Interaction Network Analysis

To explore potential interacting partners of ZmMTP1 proteins, a protein–protein interaction (PPI) network was predicted using the STRING database based on Arabidopsis homologs. The interaction network was visualized using Cytoscape software (Version 3.9.1, Cytoscape Consortium, San Diego, CA, USA). This analysis was used to provide supportive evidence for the potential involvement of ZmMTP1 proteins in metal transport and stress-response networks.

### 2.5. Cloning of ZmMTP1-1 and ZmMTP1-2 Genes and Vector Construction

The full-length coding sequences of *ZmMTP1-1* and *ZmMTP1-2* were amplified from maize cDNA using gene-specific primers. The amplified fragments were inserted into the pCAMBIA1300-211 vector under the control of the CaMV 35S promoter using seamless cloning. All constructs were verified by PCR and sequencing prior to plant transformation.

### 2.6. Generation and Identification of Transgenic Arabidopsis Lines

*Arabidopsis thaliana* ecotype Columbia-0 (Col-0) was transformed with pCAMBIA1300-211-ZmMTP1-1 and pCAMBIA1300-211-ZmMTP1-2 constructs via the floral dip method [13]. Transgenic seedlings were selected on half-strength Murashige and Skoog (1/2 MS) medium containing 30 μg·mL^−1^ hygromycin. Homozygous T_3_ lines with high transgene expression levels were identified by RT-PCR and qRT-PCR and used for subsequent experiments.

### 2.7. Cd and Zn Stress Treatments and Phenotypic Analysis in Arabidopsis

For germination assays, seeds of wild-type (WT) and transgenic Arabidopsis lines were surface-sterilized and sown on 1/2 MS agar medium supplemented with 100 or 200 μmol·L^−1^ CdCl_2_, or 150 or 300 μmol·L^−1^ ZnSO_4_. Germination rates were recorded after 7 days. For root growth assays, primary root lengths were measured after 14 days of growth. For biomass analysis, 14-day-old seedlings were transferred to soil and subjected to Cd stress by irrigating roots with 100 μmol·L^−1^ CdCl_2_ every 3 days for 7 days. Fresh weights were measured after treatment.

### 2.8. Determination of Oxidative Stress Markers and Antioxidant Enzyme Activities

Leaves from WT and transgenic Arabidopsis plants subjected to Cd stress were harvested for physiological analyses. Malondialdehyde (MDA) content and superoxide anion ($O_{2}^{\cdot -}$) levels were measured using commercial assay kits. Activities of superoxide dismutase (SOD), catalase (CAT), and peroxidase (POD) were determined following the manufacturer’s protocols. Three biological replicates were analyzed for each treatment.

### 2.9. Expression Analysis of Metal Transporter Genes in Arabidopsis

To investigate the effects of ZmMTP1 overexpression on metal transporter gene expression, WT and transgenic Arabidopsis seedlings were treated with 100 μmol·L^−1^ CdCl_2_ for 12 h. Total RNA was extracted from leaves, and qRT-PCR was performed as described above. Transcript levels of *AtHMA2*, *AtZIP4*, *AtNRAMP1*, *AtPDR8*, *AtMTP1*, and *AtPCR1* were quantified. *AtACTIN* was used as the internal reference gene.

### 2.10. Yeast Heterologous Expression and Cd Tolerance Assays

The coding sequence of *ZmMTP1-1* was cloned into the yeast expression vector pYES2. The construct and empty vector were transformed into Saccharomyces cerevisiae strain INVSc1. Transformed yeast cells were cultured in selective medium and induced with galactose. Cd tolerance was assessed by growth curve analysis in liquid medium supplemented with 20 μmol·L^−1^ CdCl_2_ and by spot assays on solid medium containing different Cd concentrations.

### 2.11. Statistical Analysis

All data are presented as mean ± standard error (SE) from three biological replicates. Statistical significance between treatments was assessed using Student’s *t*-tests and one-way ANOVA followed by Tukey’s multiple comparisons test. Differences were considered statistically significant at *p* < 0.05. All statistical computations and data visualizations were executed using Origin Pro2021 software.

## 3. Results

### 3.1. Expression Patterns of ZmMTPs Under Cd Stress in Maize

**Figure 1:**
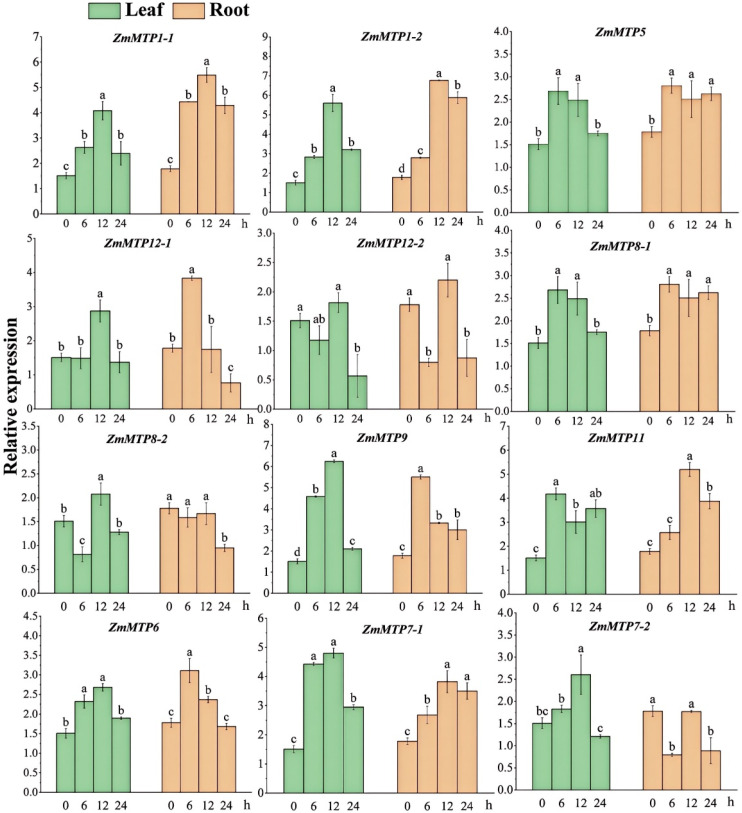
Expression patterns of *ZmMTP* genes in maize roots and leaves under Cd stress. Relative transcript levels of *ZmMTP* genes in maize roots and leaves following CdCl_2_ treatment for 0, 6, 12, and 24 h, as determined by qRT-PCR. Green and orange bars indicate expression levels in leaves and roots, respectively. Data represent means ± SE of three biological replicates. Statistical significance was analyzed using one-way ANOVA, and different letters indicate significant differences (*p* < 0.05).

Several *ZmMTP* genes exhibited moderate or transient induction following Cd exposure. For example, *ZmMTP5* and *ZmMTP12-1/12-2* (Zn-CDF subfamily) showed mild upregulation in roots or leaves at specific time points (Figure 1). Members of the Zn/Fe-CDF subfamily (*ZmMTP6*, *ZmMTP7-1*, and *ZmMTP7-2*) displayed variable but generally moderate expression changes in response to Cd treatment. In the Mn-CDF subfamily (Supplementary Figure S1), *ZmMTP8-1*, *ZmMTP8-2*, *ZmMTP9*, and *ZmMTP11* also responded to Cd stress, particularly in leaves, although their induction levels were relatively weak or transient (Figure 1).

Among all analyzed *ZmMTP* genes, *ZmMTP1-1* and *ZmMTP1-2* showed the strongest transcriptional responses to Cd stress (Figure 1). In roots, *ZmMTP1-1* transcript levels increased significantly after Cd treatment, reaching a maximum at 12 h and declining slightly at 24 h. A similar temporal pattern was observed for *ZmMTP1-2*, with a markedly higher magnitude of induction, particularly at 12 h. In leaves, both genes were also upregulated by Cd exposure, with *ZmMTP1-2* maintaining higher expression levels from 6 to 12 h before decreasing at 24 h. Although multiple ZmMTP members respond to Cd stress, *ZmMTP1-1* and *ZmMTP1-2*—both belonging to the Zn-CDF subfamily—showed the strongest. Based on their prominent Cd-responsive expression profiles and phylogenetic classification as MTP1 homologs, *ZmMTP1-1* and *ZmMTP1-2* were selected for subsequent functional characterization.

### 3.2. ZmMTP1-1 and ZmMTP1-2 Display Distinct Gene Features, Phylogenetic Relationships, and Protein Interaction Profiles

**Figure 2:**
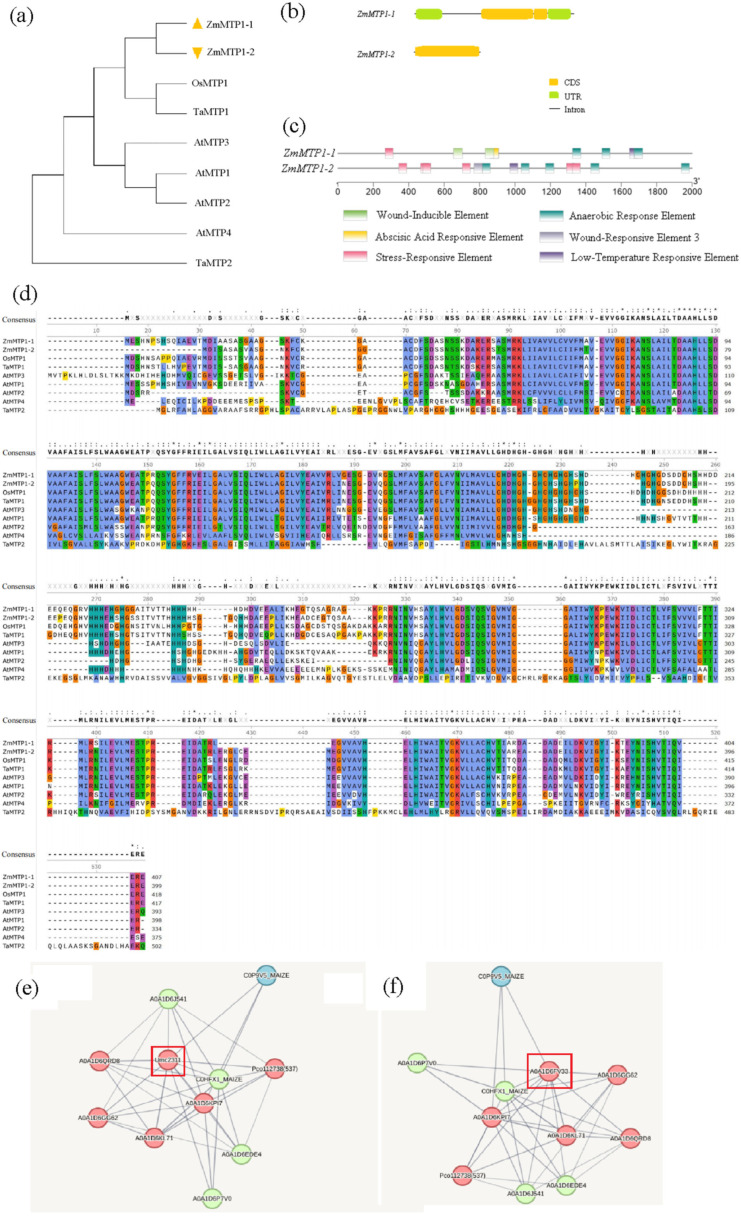
Bioinformatic characterization of *ZmMTP1-1* and *ZmMTP1-2*. (**a**) Gene structure organization of *ZmMTP1-1* and *ZmMTP1-2*. (**b**) Phylogenetic analysis of ZmMTP1-1 and ZmMTP1-2 together with closely related MTP from other plant species. The phylogenetic tree was constructed using the Neighbor-Joining method based on Clustal Omega-aligned amino acid sequences. Evolutionary distances are indicated on the branches. (**c**) Distribution of predicted cis-regulatory elements in the promoter regions of *ZmMTP1-1* and *ZmMTP1-2*. (**d**) Multiple sequence alignment of ZmMTP1-1, ZmMTP1-2, and representative MTP1 proteins. Conserved residues are shown in black, and amino acids are color-coded according to their physicochemical properties. (**e**,**f**) Predicted protein–protein interaction networks centered on ZmMTP1-1 (**e**) and ZmMTP1-2 (**f**). Nodes represent interacting proteins and are color coded by functional category. Node size reflects degree centrality, and edges indicate predicted interactions. The red boxes highlight the core ZmMTP1-1 and ZmMTP1-2 proteins, respectively, which serve as the central nodes of each interaction network.

Phylogenetic analysis showed that *ZmMTP1-1* and *ZmMTP1-2* clustered within the MTP1 subgroup and grouped closely with MTP1 proteins from other cereal crops, including *Oryza sativa* (*OsMTP1*) and *Triticum aestivum* (*TaMTP1*) (Figure 2a). Both maize proteins were also closely related to *A. thaliana AtMTP1*, supporting their classification as MTP1 homologs. Gene structure analysis revealed clear differences between the two maize genes. *ZmMTP1-1* contained two exons separated by a single intron, whereas *ZmMTP1-2* lacked introns and consisted of a single exon (Figure 2b). Protein sequence alignment showed that *OsMTP1* and *TaMTP1* shared more than 90% amino acid identity, whereas *ZmMTP1-1* and *ZmMTP1-2* exhibited approximately 55% sequence similarity (Figure 2d). Promoter analysis identified multiple cis-acting regulatory elements in both *ZmMTP1-1* and *ZmMTP1-2*, including stress-responsive and hormone-responsive elements (Figure 2c). Differences in the composition and distribution of these elements were observed between the two promoters. Protein–protein interaction networks were predicted using the STRING database. Each network contained ten predicted interaction nodes (Figure 2e,f; Supplementary Table S3). Both *ZmMTP1-1* and *ZmMTP1-2* were predicted to interact with proteins involved in zinc transport and homeostasis, including zinc transporter ZTP29 and ZIP-like protein 1. Additional predicted interactors included metal tolerance proteins and cation efflux family proteins.

### 3.3. Overexpression of ZmMTP1-1 and ZmMTP1-2 Plants Enhances Cd Stress in Arabidopsis

To examine the functional roles of *ZmMTP1-1* and *ZmMTP1-2* under metal stress conditions, transgenic *Arabidopsis thaliana* lines overexpressing each gene were generated. RT-PCR and qRT-PCR analyses confirmed the expression of *ZmMTP1-1* in three independent lines (*MTP1-1-OE1*, *MTP1-1-OE2*, and *MTP1-1*-OE3) and *ZmMTP1-2* in three independent lines (*MTP1-2*-OE1, *MTP1-2*-OE2, and *MTP1-2*-OE3) (Supplementary Figure S2a). Among these, *MTP1-1*-OE3 and *MTP1-2*-OE2 exhibited the highest transcript levels and were selected for further analysis (Supplementary Figure S2b).

**Figure 3:**
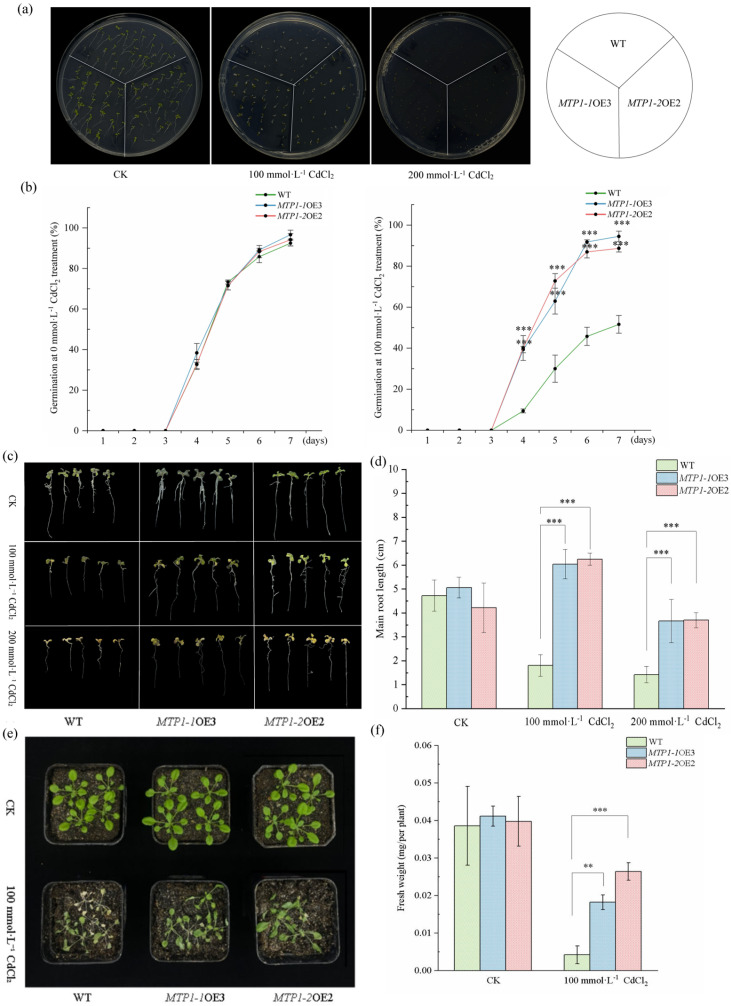
Overexpression of *ZmMTP1-1* and *ZmMTP1-2* enhances Cd tolerance in *A. thaliana.* (**a**) Phenotypes of wild-type (WT) and transgenic Arabidopsis seedlings grown on 1/2 MS medium supplemented with 0, 100, or 200 mmol·L^−1^ CdCl_2_ for 7 days. (**b**) Seed germination rates of WT, *MTP1-1*-OE3 and *MTP1-2*-OE under Cd treatments shown by green, blue and red lines. (**c**) Seedling growth of WT and transgenic plants after transfer from 1/2 MS medium to Cd-containing medium. (**d**) Primary root lengths of seedlings shown in (**c**). (**e**) Phenotypes of soil-grown WT and transgenic plants treated with or without 100 μmol·L^−1^ CdCl_2._ (**f**) Fresh weights of plants shown in (**e**). Data represent means ± SE of three independent experiments. Statistical significance was determined by one-way ANOVA followed by Tukey’s post hoc test. Asterisks indicate significant differences compared with the wild-type (WT) control: ** *p* < 0.01, *** *p* < 0.001. Scale bars = 1 cm.

Under control conditions, no obvious differences were observed between wild-type (WT) and transgenic plants in terms of seed germination, primary root growth, or overall morphology (Figure 3). Under Cd stress, clear phenotypic differences were observed between WT and transgenic plants. On 1/2 MS medium supplemented with 100 mmol·L^−1^ CdCl_2_, both *MTP1-1*-OE3 and *MTP1-2*-OE2 showed higher germination rates than WT plants (Figure 3a,b). At 200 mmol·L^−1^ CdCl_2_, seed germination was reduced in all genotypes; however, transgenic seedlings exhibited improved growth compared with WT (Figure 3a). Root growth assays showed that primary root lengths were comparable among all genotypes under control conditions (Figure 3c,d). After treatment with 100 mmol·L^−1^ CdCl_2_, *MTP1-1*-OE3 and *MTP1-2*-OE2 maintained significantly longer primary roots than WT plants. This difference remained evident under 200 mmol·L^−1^ CdCl_2_ (Figure 3d). In soil-grown plants, WT seedlings exhibited severe growth inhibition after 7 days of Cd treatment, whereas MTP1-1OE3 and MTP1-2OE2 maintained better growth status and higher survival rates (Figure 3e). Consistently, fresh weight measurements showed significantly higher biomass in transgenic plants than in WT under Cd stress (Figure 3f).

Under ZnSO_4_ treatment, no significant differences were observed among WT and transgenic lines under control conditions (Supplementary Figure S3a–d). At 150 μmol·L^−1^ ZnSO_4_, germination rates were comparable among genotypes, whereas at 300 μmol·L^−1^ ZnSO_4_, *MTP1-1*-OE3 and *MTP1-2*-OE2 showed higher germination rates than WT plants (Supplementary Figure S3b). Root elongation was inhibited by Zn stress in WT plants, while transgenic lines maintained longer primary roots, particularly at 300 μmol·L^−1^ ZnSO_4_ (Supplementary Figure S3c,d).

### 3.4. ZmMTP1-1 and ZmMTP1-2 Positively Regulate Antioxidant Defense and Reduce Oxidative Damage Under Cd Stress

**Figure 4:**
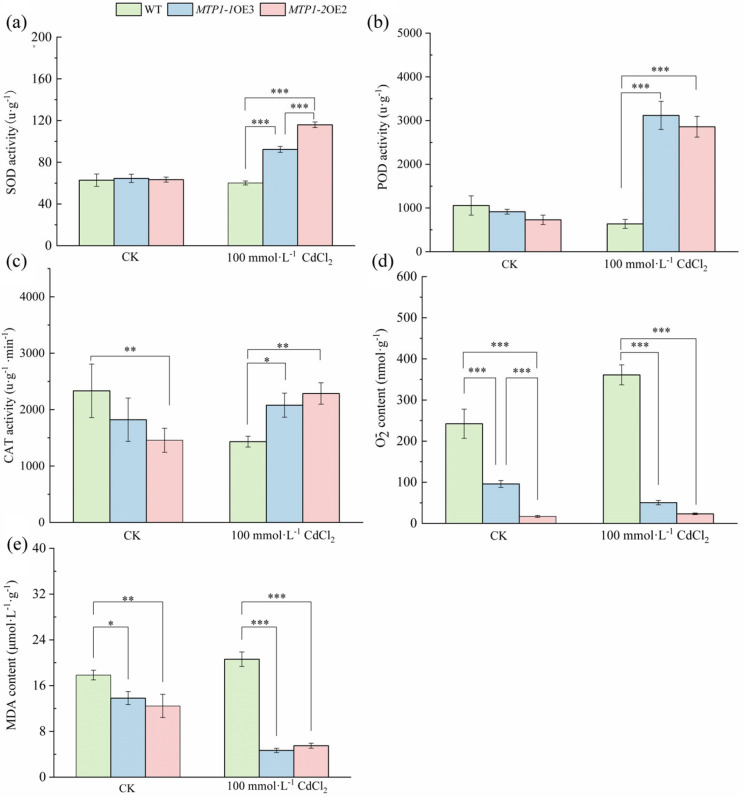
Effects of *ZmMTP1-1* and *ZmMTP1-2* overexpression on antioxidant responses under Cd stress. Activities of antioxidant enzymes and oxidative stress indicators were measured in WT and transgenic Arabidopsis plants under control conditions or 100 mmol·L^−1^ CdCl_2_ treatment. (**a**) Superoxide dismutase (SOD) activity. (**b**) Peroxidase (POD) activity. (**c**) Catalase (CAT) activity. (**d**) Superoxide anion ($O_{2}^{\cdot -}$) content. (**e**) Malondialdehyde (MDA) content. Data represent means ± SE of three independent experiments. Statistical significance was determined by one-way ANOVA followed by Tukey’s post hoc test. Asterisks indicate significant differences compared with the wild-type (WT) control: * *p* < 0.05, ** *p* < 0.01, *** *p* < 0.001.

The activities of antioxidant enzymes and indicators of oxidative damage were examined in WT and transgenic Arabidopsis plants under Cd stress. Under control conditions, no significant differences in SOD, POD, or CAT activities were detected between WT and transgenic lines (Figure 4a–c). Following treatment with 100 mmol·L^−1^ CdCl_2_, SOD activity increased in both *MTP1-1*-OE3 and *MTP1-2*-OE2 plants, with values significantly higher than those observed in WT (Figure 4a). POD activity was also markedly elevated in transgenic lines under Cd stress (Figure 4b). Similarly, CAT activity increased significantly in *MTP1-1*-OE3 and *MTP1-2*-OE2 compared with WT plants (Figure 4c). Measurements of oxidative damage indicators showed reduced accumulation of $O_{2}^{\cdot -}$ in transgenic plants relative to WT under Cd stress (Figure 4d). MDA content was also significantly lower in *MTP1-1*-OE3 and *MTP1-2*-OE2 plants than in WT following Cd exposure (Figure 4e).

### 3.5. Upregulation of Metal Transporter and Cation Exchanger Genes in ZmMTP1-1- and ZmMTP1-2-Overexpressing Arabidopsis Under Cd Stress

**Figure 5:**
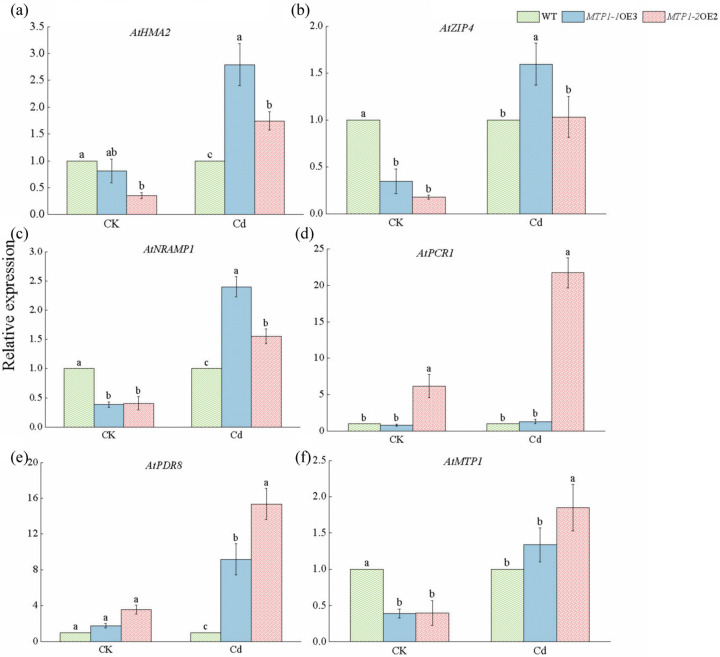
Expression of metal transporter genes in WT and *ZmMTP1-1*- and *ZmMTP1-2*-overexpressing Arabidopsis under Cd stress. Relative transcript levels of endogenous metal transporter genes in WT and transgenic plants treated with or without 100 mmol·L^−1^ CdCl_2_. (**a**) *AtHMA2*, (**b**) *AtZIP4*, (**c**) *AtNRAMP1*, (**d**) *AtPCR1*, (**e**) *AtPDR8*, and (**f**) *AtMTP1*. Values represent means ± SE of three biological replicates. Statistical significance was determined by one-way ANOVA, and different letters indicate significant differences (*p* < 0.05). The Relative transcript levels of WT, *MTP1-1*-OE3 and *MTP1-2*-OE under Cd treatments shown by green, blue and red.

The transcript levels of several endogenous metal transporter and cation exchanger genes were examined in WT and transgenic plants. Under control conditions, expression levels of *AtHMA2*, *AtZIP4*, *AtNRAMP1*, *AtPCR1*, *AtPDR8*, and *AtMTP1* were similar among all genotypes (Figure 5a–f). After CdCl_2_ treatment, *AtHMA2* transcript levels increased in both MTP1-1OE3 and MTP1-2OE2 plants compared with WT (Figure 5a). *AtZIP4* and *AtNRAMP1* were also upregulated in transgenic lines under Cd stress (Figure 5b,c). In contrast, *AtPCR1* expression increased markedly in *MTP1-2*-OE2 plants, while no significant difference was detected between *MTP1-1*-OE3 and WT (Figure 5d). Expression of *AtPDR8* increased in both transgenic lines under Cd treatment, with higher transcript levels in *MTP1-2*OE2 plants (Figure 5e). *AtMTP1* expression was also elevated in the *MTP1-1*-OE3 line, whereas *MTP1-2*-OE2 showed an increasing trend without statistical significance (Figure 5f).

### 3.6. Heterologous Expression of ZmMTP1-1 Enhanced Cd Tolerance in Yeast

**Figure 6:**
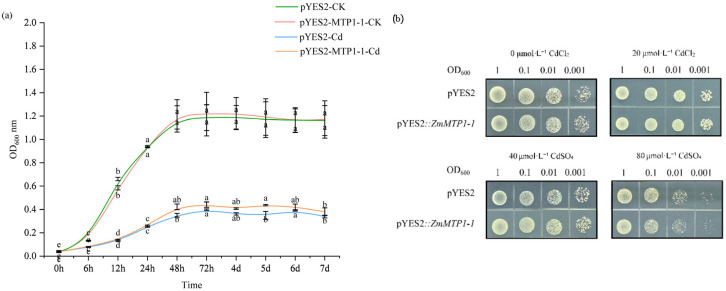
Heterologous expression of *ZmMTP1-1* enhances Cd tolerance in yeast. (**a**) Growth curves of yeast cells carrying the empty vector (pYES2) or expressing *ZmMTP1-1* (pYES2::ZmMTP1-1) cultured in liquid medium supplemented with different concentrations of CdCl_2_. (**b**) Spot assays of yeast strains grown on solid medium containing various concentrations of CdSO_4_. Serial dilutions were plated and incubated to assess Cd tolerance. Statistical significance was determined by one-way ANOVA, and different letters indicate significant differences (*p* < 0.05).

To assess the function of *ZmMTP1-1* in a heterologous system, the gene was expressed in yeast using the pYES2 vector. Under Cd-free conditions, yeast cells carrying pYES2 or pYES2::*ZmMTP1-1* exhibited similar growth kinetics, with comparable OD_600_ values throughout the cultivation period (Figure 6a). Under Cd stress, growth differences between the two strains became evident. In the presence of 20 μmol·L^−1^ CdCl_2_, pYES2::*ZmMTP1-1* cells maintained higher OD_600_ values than pYES2 control cells from 24 h onward (Figure 6a). This difference increased at later time points and persisted throughout the 7-day observation period. Spot assays on solid medium showed no visible differences between the two strains under control conditions (Figure 6b). Under increasing Cd concentrations (40, and 80 μmol·L^−1^ CdSO_4_), yeast cells expressing *ZmMTP1-1* exhibited stronger growth across serial dilutions compared with the empty vector control (Figure 6b).

## 4. Discussion

Cd contamination poses a serious threat to crop productivity and food safety, particularly in maize, a staple cereal crop characterized by relatively high metal uptake and translocation capacities [14]. Elucidating the molecular mechanisms underlying Cd tolerance is therefore essential for developing sustainable strategies to reduce Cd accumulation in maize [15]. In the present study, two maize MTP1 homologs, *ZmMTP1-1* and *ZmMTP1-2*, were systematically characterized through integrated bioinformatic analyses, expression profiling, and functional validation in *Arabidopsis* and yeast. The results demonstrate that these two transporters participate in Cd and Zn tolerance and suggest that they function within a coordinated detoxification framework involving metal transport, antioxidant defense, and transcriptional regulation.

Although multiple *ZmMTP* genes responded transcriptionally to Cd stress, the prioritization of *ZmMTP1-1* and *ZmMTP1-2* for further functional characterization was based on a combination of phylogenetic position, expression magnitude, and response dynamics. Phylogenetic analyses placed both genes within the Zn-CDF subfamily and clustered them with well-characterized MTP1 homologs from *Arabidopsis*, rice, and wheat (Supplementary Figure S1), which are known to function as key Zn/Cd transporters involved in metal sequestration and detoxification. This evolutionary conservation suggests that *ZmMTP1-1* and *ZmMTP1-2* are more likely to play direct roles in Cd tolerance than members of other MTP subfamilies. Consistently, qRT-PCR analysis of the complete *ZmMTP* gene family revealed that *ZmMTP1-1* and *ZmMTP1-2* exhibited the strongest and most sustained transcriptional induction under Cd stress, particularly in roots, the primary site of Cd uptake. In contrast, although several Zn/Fe-CDF and Mn-CDF members, such as *ZmMTP6*, *ZmMTP8*, *ZmMTP9*, and *ZmMTP11*, displayed moderate or transient Cd-responsive expression, their responses were generally weaker, less persistent, or more tissue-restricted. These observations are consistent with previous reports indicating that Zn-CDF transporters play central roles not only in micronutrient homeostasis but also in plant responses to environmental stresses [16].

Notably, *ZmMTP1-2* exhibited a higher induction amplitude and more sustained expression than *ZmMTP1-1* in both roots and leaves, suggesting potential functional divergence between the two paralogs. Such differential expression dynamics imply that *ZmMTP1-1* and *ZmMTP1-2* may contribute to Cd tolerance through partially overlapping yet non-redundant mechanisms. Structural differences between the two proteins, including distinct exon–intron organizations and variation in the histidine-rich loop, may underlie their functional differentiation. The histidine-rich region has been reported to influence metal-binding affinity and substrate specificity in CDF transporters [17]. In this context, the extended histidine-rich region of *ZmMTP1-2* may contribute to its stronger transcriptional response and enhanced Cd tolerance observed in heterologous systems. In addition, promoter analyses revealed an abundance of stress- and hormone-responsive cis-elements, including motifs associated with ABA, MeJA, and drought responses, providing a regulatory basis for the dynamic expression of *ZmMTP1-1* and *ZmMTP1-2* under abiotic stresses [18].

Functional analyses in transgenic Arabidopsis demonstrated that overexpression of *ZmMTP1-1* and *ZmMTP1-2* enhanced tolerance to both Cd and Zn without affecting normal growth under control conditions. Transgenic plants exhibited higher germination rates, longer primary roots, and increased biomass accumulation under Cd stress compared with wild-type plants. The growth advantage conferred by *ZmMTP1-2* overexpression was more pronounced than that observed for *ZmMTP1-1*, consistent with its stronger transcriptional induction and distinct structural features. Compared with previous studies on *OsMTP1* and *AtMTP1* [9,10], maize MTP1 homologs conferred comparable or greater levels of Cd tolerance, suggesting potential species-specific functional optimization. Such differences may reflect adaptive evolution of maize in environments characterized by higher transpiration rates and enhanced metal uptake capacity [12]. The conserved Cd tolerance observed upon heterologous expression of *ZmMTP1-1* in yeast further supports the intrinsic detoxification function of MTP1 proteins and highlights the evolutionary conservation of their roles in metal homeostasis [19].

Beyond their role in metal transport, overexpression of *ZmMTP1-1* and *ZmMTP1-2* markedly enhanced antioxidant defense capacity in Arabidopsis under Cd stress. Elevated activities of SOD, CAT, and POD, together with reduced accumulation of malondialdehyde and superoxide anions, indicate effective mitigation of Cd-induced oxidative damage. Cd stress is well known to induce excessive reactive oxygen species (ROS) production, leading to membrane damage and impaired cellular function [4]. Strengthening antioxidant capacity therefore represents an important component of Cd tolerance [10].

In addition, overexpression of *ZmMTP1-1* and *ZmMTP1-2* led to the upregulation of multiple endogenous metal transporter genes, including *AtHMA2*, *AtZIP4*, *AtNRAMP1*, *AtPDR8*, and *AtMTP1*. These transporters function cooperatively in metal efflux, compartmentalization, and long-distance transport [20,21,22]. The coordinated transcriptional activation of these genes supports a model in which ZmMTP1 proteins act as components of an integrated metal detoxification network rather than functioning independently. This coordination was more pronounced under Cd stress than under Zn stress, consistent with the higher toxicity and non-essential nature of Cd relative to Zn [1].

The relatively high expression of *ZmMTP1-1* and *ZmMTP1-2* genes in reproductive tissues further raises the possibility that these transporters may contribute to limiting Cd accumulation in grains, an important consideration for improving food safety. Although heterologous systems provide strong evidence for conserved MTP1 detoxification functions, recent advances in genome editing and transformation technologies [23]. now enable more precise functional validation in maize. Future studies using CRISPR-based knockout lines and overexpression lines in maize will be essential to definitively establish their physiological roles in maize.

## 5. Conclusions

In summary, this study identifies *ZmMTP1-1* and *ZmMTP1-2* as two Cd-responsive MTP1 homologs that contribute to heavy metal tolerance in maize. Both genes belong to the Zn-CDF subfamily and exhibit strong transcriptional induction under Cd stress, particularly in roots. Functional analyses in *Arabidopsis thaliana* and yeast demonstrate that overexpression of *ZmMTP1-1* and *ZmMTP1-2* enhances tolerance to Cd and Zn without impairing normal growth. Enhanced metal tolerance is associated with improved antioxidant capacity and coordinated regulation of metal transporter genes, suggesting that ZmMTP1 proteins participate in an integrated metal detoxification network. These findings advance our understanding of MTP-mediated metal homeostasis in maize and provide candidate genes for future efforts aimed at improving heavy metal tolerance and crop safety in contaminated soils.

## Data Availability

Data will be made available on request.

## Supplementary Materials

The following supporting information can be downloaded at: https://www.mdpi.com/article/10.3390/plants15060941/s1, Figure S1. Phylogenetic analysis of MTPs from four plant species; Figure S2. Identification of ZmMTP1-1 and ZmMTP1-2 overexpression lines in Arabidopsis; Figure S3. Effects of Zn stress on WT, ZmMTP1-1 and ZmMTP1-2-overexpressing Arabidopsis plants; Table S1. Primers used for qRT-PCR analysis in this study; Table S2. Physicochemical properties of ZmMTP1-1 and ZmMTP1-2 proteins; Table S3. Predicted protein–protein interaction partners of ZmMTP1-1 and ZmMTP1-2.
