# Supplementary material for: Functional Characterization of Maize ZmMTP1-1 and ZmMTP1-2 Reveals Their Roles in Cd Tolerance
Source: Plants (Basel). 2026 Mar 19;15(6):941. doi: 10.3390/plants15060941 (PMC13030030; doi:10.3390/plants15060941)
Supplement: Supplementary file 1 [file plants-15-00941-s001.zip › plants-4164827 - supplemental files.pdf]

Supplementary Tables

Table S1. Primers used for qRT-PCR analysis in this study.

Gene Name	Gene ID	Primer Sequence of F	Primer Sequence of R	Function
ZmMTP9	*Zm00001d040002*	5'-ATACTCCTTCGTCGGATGACA-3'	5'-TAACGGCGACATACGGCTA-3'	qRT-PCR
ZmMTP11	*Zm00001d042939*	5'-GACCAGAACCGTGCCACA-3'	5'-CACGTCTCCGAGCATCGATT-3'	
ZmMTP8-1	*Zm00001d028344*	5'ACCATAAGCCGGAGCATT-3'	5'-ATTCTGCACGACAATCTACCAC-3'	
ZmMTP8-2	*Zm0000ld018242*	5'-CTCCAGATATGCTGCAGTTGCT-3'	5'-CTCAGCCGCATATCTTCCG-3'	
ZmMTP6	*Zm00001d029129*	5'-GCGCTCGAACATCTAATGCTG-3'	5'-ACTTCCATTGCATCCCGAA-3'	
ZmMTP7-2	*Zm00001d004091*	5'-CTATCTCATGGAGCCGGTCAC-3'	5'-ATTGCAGCTTCCCGGAAC-3'	
ZmMTP7-1	*Zm00001d025130*	5'-GCAATGTCCAAGGATGATACGGA-3'	5'-ATCTCAGACTCCAGCCGAT-3'	
ZmMTP5	*Zm00001d051830*	5'-ATATCCTACTGCAAATCGCACCT-3'	5'-CTCCCAGAATCGACCTTGCC-3'	
ZmMTP12-1	*Zm00001d032262*	5'-GAGCAGTCCCGATCAGCC-3'	5'-AGTGCATAAGAACGTCATTGCAAA-3'	
ZmMTP12-2	*Zm00001d050335*	5'-TTGGGCTGATCTCTGATGCTT-3'	5'-CCTGCCCCTTCCAAAGTTGT-3'	
ZmMTP1-2	*Zm00001d010954*	5'-CCACTCAGGCACTGGACAACA-3'	5'-CCAAGCACATGAAGGTAAGCG-3'	
ZmMTP1-1	*Zm00001d035447*	5'-GGTACATCAAGACGGAGTACAACA-3'	5'-CACCACCGAGCACACCAGA-3'	
ZmMTP 1-1	*Zm00001d035447*	5'-ATCCGCTCTTCTCCCTCCCTT-3'	5'-GAGCACACCAGAGCTCGAATG-3'	Cloning of the full-length CDS sequence of *ZmMTP1-1*
ZmMTP 1-2	*Zm00001d010954*	5'-GCCTCGCTCAATCGATGGAA-3'	5'-CCTCTATTCACGCTCGACCTG-3'	Cloning of the full-length CDS sequence of *ZmMTP1-2*
HindIII 1-1	*Zm00001d035447*	5'-CAAATCGACTCTAGTCTAGAAAGCTT	5'-GCCCTTGCTCACCATGGTACC	Seamless cloning
KpnI 1-1		ATCCGCTCTTCTCCCTCCCTT-3'	GCGCCAGCCATTTATGCCCAA-3'	
HindIII 1-2	*Zm00001d01095*	5'-TCGACTCTAGTCTAGAAAGCTT	5'-GCCCTTGCTCACCATGGTACC	

Gene Name	Gene ID	Primer Sequence of F	Primer Sequence of R	Function
KpnI 1-2	*4*	GCCTCGCTCAATCGATGGAA-3’	CCTCTATTCACGCTCGACCTG-3’	
AtMTP1	*AT2G46800*	5’-CTAGGCAGACTTACGGGTTC-3’	5’-CTGTAACAATTCTGATAATCGCTT-3’	qRT-PCR
AtZIP4	*ATIG10970*	5’-GGATACTGGACTCGCTGTCA-3’	5’-GCCTCAAATTACAACTCATCCTC-3’	
AtHMA2	AT4G30110	5’-TCAAGAGAAAGCGATGAAACC-3’	5’-CACAACCGTTACATTGTCT-3’	
AtNRAMP1	ATIG80830	5’-TGGCCTTTCTCATAAACGTC-3’	5’-CGAAGCCTTGTTTAAGTCCAA-3’	
AtPCR1	ATIG14880	5’-CTGGAGCATTATACATGTTGA-3’	5’-TATTGTTGGGTCAAAGCAC-3’	
AtPDR8	ATIG59870	5’-ACCATTCATCAGCCTAGCA-3’	5’-CCTTGTGAGAGTTTTGACCCA-3’	
ZmActin	Zm00001d010159	5’-CGAAGCCTTGTTTAAGTCCAA-3’	5’-AACGATTCCTGGACCTGCCTC-3’	Reference genes of qRT-PCR
AtActin	AT3G18780	5’-CGAAGCCTTGTTTAAGTCCAA-3’	5’-AACGATTCCTGGACCTGCCTC-3’	

Table S2. Physicochemical properties of *ZmMTP1-1* and *ZmMTP1-2* proteins.

Protein ID	MTP1 Protein Name	Domain	Chromosomal Locations	Amino Acid	MW/KD	pI	Subcellular Localization	Transmembr Domains
NP_001130946.1	ZmMTP1-1	Cation_efflux	6	407	44.06	6.04	Vacuole	6
NP_001335895.1	ZmMTP1-2	Cation_efflux	8	399	43.62	5.89	Vacuole	6
NP_001407014.1	OsMTP1	Cation_efflux	5	418	45.58	5.87	Vacuole	6
XP_044458137.1	TaMTP1	Cation_efflux	1	417	45.47	6.02	Vacuole	6
XP_044363486.1	TaMTP2	Cation_efflux<br>ZT_dimer	4	502	53.81	8.26	Vacuole	0
NP_001318436.1	AtMTP1	Cation_efflux	2	398	43.83	6.13	Vacuole	6
NP_191753.1	AtMTP2	Cation_efflux	3	334	37.34	6.00	Vacuole	6
NP_191440.2	AtMTP3	Cation_efflux	3	393	42.93	5.89	Vacuole	6
NP_180502.2	AtMTP4	Cation_efflux	2	375	42.34	6.13	Vacuole	5

Table S3. Predicted protein–protein interaction partners of ZmMTP1-1 and ZmMTP1-2.

Protein ID	Protein Name	Function
A0A1D6KL71	IAA-alanine resistance protein 1	Involved in resistance to auxin conjugates, particularly IAA-alanine, helping regulate auxin levels and plant growth.
A0A1D6GG62	Zinc transporter ZTP29.	Functions as a zinc transporter, maintaining zinc homeostasis by transporting zinc ions across cellular membranes.
A0A1D6QRD8	ZIP-lke protein 1	Functions as a zinc transporter, maintaining zinc homeostasis by transporting zinc ions across cellular membranes.
Pco112738(537)	Putative zinc transporter	Acts as a putative zinc transporter, likely involved in zinc ion transport and homeostasis.
A0A1D6P7V0	5-hydroxyisourate hydrolase	Involved in the degradation of purines, catalyzing the hydrolysis of 5-hydroxyisourate, a product of uric acid breakdown.
A0A1D6KP17	Cation effux family protein	Involved in the degradation of purines, catalyzing the hydrolysis of 5-hydroxyisourate, a product of uric acid breakdown.
COHFX1_MAIZE	Cation effuxfamily protein	Member of the cation efflux family, involved in transporting metal ions (e.g., zinc) out of the cell.
COP9V5_MAIZE	Xylulose kinase-2	Catalyzes the phosphorylation of xylulose, playing a key role in the pentose phosphate pathway and carbohydrate metabolism.
A0A106EDE4	Metal tolerance protein C4	Catalyzes the phosphorylation of xylulose, playing a key role in the pentose phosphate pathway and carbohydrate metabolism.
A0A1D6J541	Metal tolerance protein C4	Catalyzes the phosphorylation of xylulose, playing a key role in the pentose phosphate pathway and carbohydrate metabolism.

Supplementary Figures

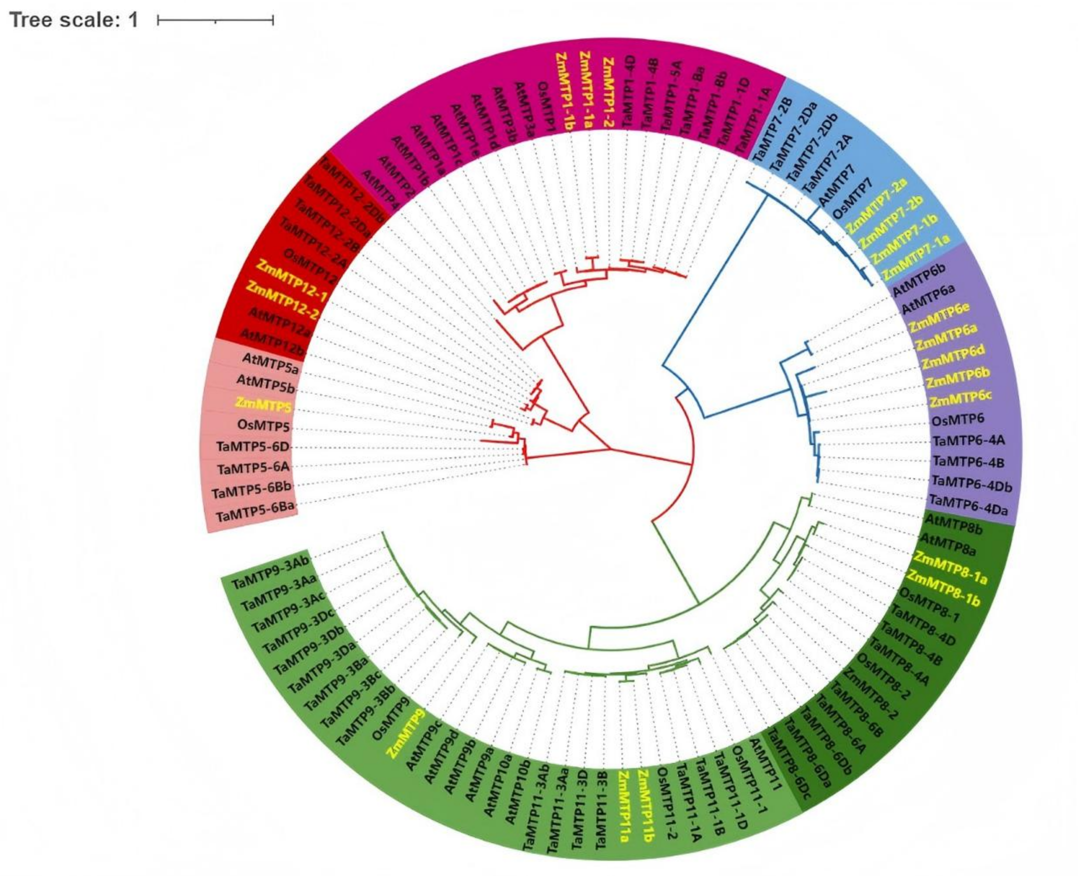

Figure S1. Phylogenetic analysis of MTP proteins from four plant species. The phylogenetic tree was constructed using the Neighbor-Joining (NJ) method based on the multiple sequence alignment of full-length amino acid sequences of MTP proteins from *Zea mays* (maize), *Arabidopsis thaliana* (thale cress), *Oryza sativa* (rice), and *Triticum aestivum* (wheat). Different color-coded branches represent the three major MTP subfamilies**:** Zn-CDF (red), Zn/Fe-CDF (blue), and Mn-CDF (green). The tree scale bar indicates a genetic distance of 1.

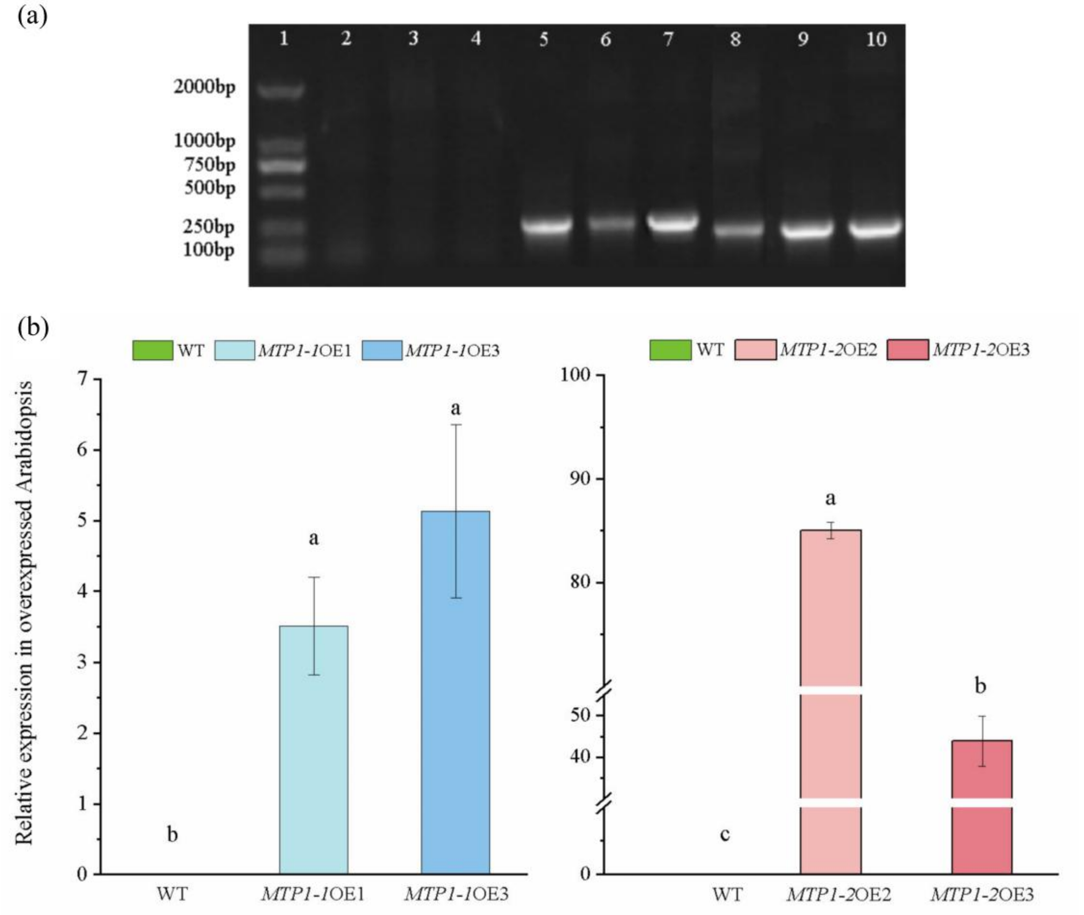

Figure S2. Molecular identification of transgenic Arabidopsis overexpressing maize *ZmMTP1-1* and *ZmMTP1-2* genes. (a) Semiquantitative RT-PCR analysis of *ZmMTP1-1* and *ZmMTP1-2* expression in wild-type (WT) and independent transgenic lines. No amplification products were detected in WT lanes (1–3), confirming the absence of endogenous MTP1 homologs in Arabidopsis. Lane 1, marker; lanes 2–4, WT; lanes 5–7, *ZmMTP1-1* overexpression (OE) lines; lanes 8–10, *ZmMTP1-2* overexpression lines. (b) Relative expression analysis of *ZmMTP1-1* and *ZmMTP1-2* in representative transgenic lines via quantitative real-time PCR (qRT-PCR). The expression levels were normalized to the *Actin* internal control. Data are presented as means ± SE from three independent biological replicates. Significant differences between groups were analyzed by one-way ANOVA followed by Duncan's post-hoc test (or Student's t-test), with significance set at $P < 0.05$.

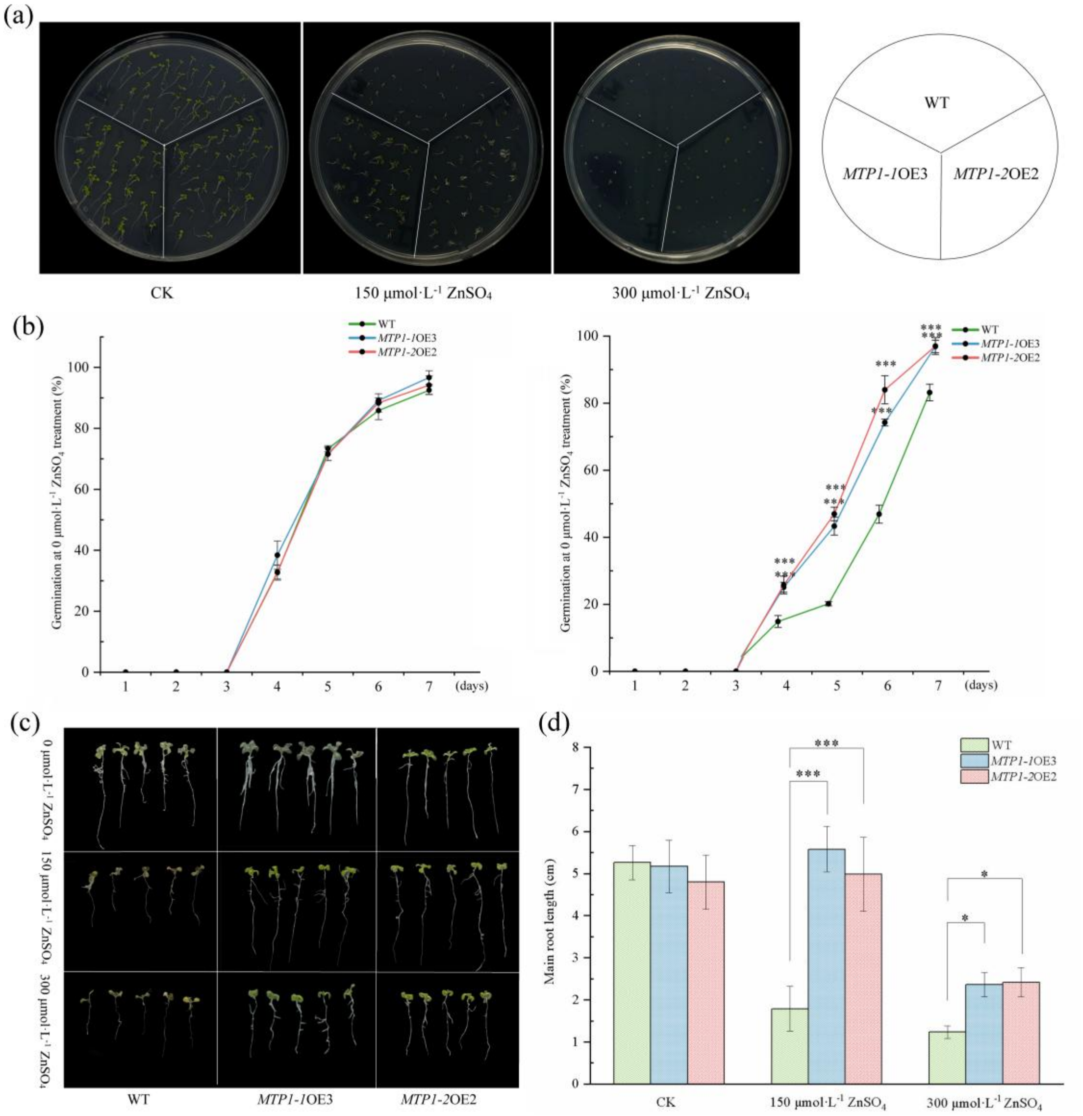

Figure S3. Effects of Zn stress on WT, *ZmMTP1-1* and *ZmMTP1-2*-overexpressing Arabidopsis plants. (a) Phenotypes of WT and transgenic seedlings grown on 1/2 MS medium supplemented with 0, 150, or 300 μmol·L^{-1} $ZnSO_4$ for 7 days.(b) Seed germination rates under Zn stress conditions.(c) Seedling growth following transfer to Zn- containing medium.(d) Primary root lengths of seedlings shown in (c).Data represent means ± SE of three independent experiments. Significant differences were analyzed by *t*-test ($p < 0.05$). Scale bars = 1 cm.
